# International medical tourism of US cancer patients for alternative cancer treatments: Financial, demographic, and clinical profiles of online crowdfunding campaigns

**DOI:** 10.1002/cam4.5636

**Published:** 2023-01-19

**Authors:** John Peterson, Trevor F. Wilson, Melissa H. Watt, Josh Gruhl, Sydney Davis, Jaxon Olsen, Matthew W. Parsons, Benjamin H. Kann, Briony Swire‐Thompson, Angela Fagerlin, Echo L. Warner, Andy J. King, Fumiko Chino, Skyler B. Johnson

**Affiliations:** ^1^ Department of Radiation Oncology University of Utah, Huntsman Cancer Institute Salt Lake City Utah USA; ^2^ Department of Population Health Sciences University of Utah Salt Lake City Utah USA; ^3^ Department of Radiation Oncology, Dana‐Farber Cancer Institute/Brigham and Women's Hospital Harvard Medical School Boston Massachusetts USA; ^4^ Network Science Institute Northeastern University Boston Massachusetts USA; ^5^ Institute for Quantitative Social Science Harvard University Cambridge Massachusetts USA; ^6^ Salt Lake City VA Informatics Decision‐Enhancement and Analytic Sciences (IDEAS) Center for Innovation Salt Lake City Utah USA; ^7^ College of Nursing University of Utah Salt Lake City Utah USA; ^8^ Cancer Control & Population Sciences Huntsman Cancer Institute Salt Lake City Utah USA; ^9^ Department of Communication University of Utah Salt Lake City Utah USA; ^10^ Department of Radiation Oncology Memorial Sloan Kettering Cancer Center New York New York USA

**Keywords:** alternative cancer therapy, financial toxicity, health misinformation, internet research, medical tourism

## Abstract

**Background:**

Previous research has found that individuals may travel outside their home countries in pursuit of alternative cancer therapies (ACT). The goal of this study is to compare individuals in the United States who propose plans for travel abroad for ACT, compared with individuals who seek ACT domestically.

**Methods:**

Clinical and treatment data were extracted from campaign descriptions of 615 GoFundMe® campaigns fundraising for individuals in the United States seeking ACT between 2011 and 2019. We examined treatment modalities, treatment location, fundraising metrics, and online engagement within campaign profiles. Clinical and demographic differences between those who proposed international travel and those who sought ACT domestically were examined using two‐sided Fisher's exact tests. Differences in financial and social engagement data were examined using two‐sided Mann–Whitney tests.

**Results:**

Of the total 615 campaigns, 237 (38.5%) mentioned plans to travel internationally for ACT, with the majority (81.9%) pursuing travel to Mexico. Campaigns that proposed international treatment requested more money ($35,000 vs. $22,650, *p < 0*.001), raised more money ($7833 vs. $5035, *p < 0*.001), had more donors (57 vs. 45, *p = 0*.02), and were shared more times (377 vs. 290.5, *p = 0*.008) compared to campaigns that did not. The median financial shortfall was greater for campaigns pursuing treatments internationally (−$22,640 vs. ‐$13,436, *p < 0*.003).

**Conclusions:**

Campaigns proposing international travel for ACT requested and received more money, were shared more online, and had more donors. However, there was significantly more unmet financial need among this group, highlighting potential financial toxicity on patients and families.

## 1 INTRODUCTION

In their 2020 annual survey, the American Society of Clinical Oncology found that approximately one in three respondents in the United States believed alternative cancer therapies (ACT) alone could cure cancer.^1^ ACT is a subcategory of complementary and alternative medicine (CAM), a broad term defined by the National Cancer Institute (NCI) to include the multitude of treatment modalities outside the medical mainstream that are used in place of conventional cancer treatments such as chemotherapy, radiotherapy, and surgery.^2^ Complementary therapies are non‐traditional therapies that are integrated into a patient's treatment plan alongside conventional therapies, such as radiotherapy and chemotherapy, often playing a supportive role by helping with symptom management and addressing a patient's physical, emotional, or spiritual health.^2^ In contrast, ACTs are used in place of the standard of care and therefore do not include treatments intended as complementary therapies.^2^ ACTs pose risks to cancer patients as research has found mortality rates among ACT users to be significantly higher than those who do not use them.^3^ Additionally, most insurance companies and public health systems often do not cover the high costs of ACT. Therefore, using ACT carries potential risks of financial toxicity, defined by the negative health outcomes associated with paying for expensive medical treatment (e.g., emotional distress, loss of employment during treatment and recovery, and medical non‐compliance).^4^ Nevertheless, ACT use is frequent worldwide, and many individuals may turn to online crowdfunding sites, including GoFundMe® (gofundme.com), to help offset the often significant personal and family costs.^5^, ^6^, ^7^, ^8^, ^9^, ^10^, ^11^

The international medical tourism industry is estimated at $100 billion and expected to reach $165.3 billion in 2023, though the market contribution of ACT remains unknown.^12^ Due to legal restrictions, many ACT modalities are not available in the US, which could prompt some individuals with cancer to travel to countries with little to no regulation to access the specific therapies they seek, resulting in some individuals with cancer traveling to countries with looser regulation in the pursuit of ACT they cannot receive domestically.^5^ Other individuals opt to use ACT available in their home countries, including home remedies, mail order supplements, and in‐person treatments. Previous research has identified specific clinics that are common treatment destinations for the beneficiaries of online fundraisers seeking ACT abroad,^5^, ^6^ but the differences between campaigns for individuals who travel internationally for ACT and those that receive treatment in their home countries have not been reported.

In this study, we aimed to determine whether individuals in the United States who planned to travel internationally for ACT differed financially and clinically from individuals who planned to seek ACT domestically. We used publicly available online crowdfunding data to better understand the financial implications associated with pursuing ACT outside of the United States and determine whether the campaigns for individuals traveling internationally for ACT differed clinically, financially, or in regards to social media engagement compared with those who did not propose international travel. Understanding the clinical and financial implications of fundraising for ACT will help clinicians target these issues in cancer patient education.

## 2 MATERIALS AND METHODS

The study was a cross‐sectional analysis of GoFundMe® campaigns created between 2011 and 2019 to help individuals with cancer pay for ACT. GoFundMe® is an online crowdfunding platform launched in 2010 that allows individuals to fundraise online for specific causes, including covering medical expenses. The site receives over $140 million donations each month.^13^ GoFundMe® allows for campaign information to be shared with others through ten forms of social media: Facebook, Twitter, email, WhatsApp, SMS text, Instagram, Slack, YouTube, TikTok, or Linktree. The number of page shares recorded for each campaign refers to the number of times the campaign has been linked to one of these 10 social media. Subsequent shares of the original link within a specific platform (e.g., retweets of the link or reshares on Facebook) are not included in this number. This study did not involve interaction or intervention with human subjects, and therefore, did not meet the definition of human subject research, resulting in an IRB exempt status.

### 2.1 Data source and Selection

Custom Python web scraping code was developed and used to search GoFundMe® for English‐language campaigns for beneficiaries living in the United States using the term “alternative cancer” covering campaigns published between 2011 and 2019 (Multimedia Appendix S1). Data were abstracted on October 25, 2019, and the query yielded exactly 1000 campaigns. Given travel restrictions imposed by the US government in response to the extraordinary circumstances of the COVID‐19 pandemic, campaigns initiated after 2019 were not included in our sample. Campaigns were reviewed manually (JP, TW, JG, SD, and JO) to assess whether it met the following inclusion criteria created a priori: (1) written in English, (2) included a campaign description, (3) raised money for ACT sought by an individual with cancer and, (4) benefitted an individual with cancer in the United States. In this study, “alternative cancer therapy” was defined using the National Cancer Institute's definition for alternative medicine: “Treatments that are used instead of standard treatments. … Alternative medicine may include special diets, megadose vitamins, herbal preparations, special teas, and magnet therapy. For example, a special diet may be used instead of anticancer drugs as a treatment for cancer.”^14^ Using this definition, each campaign was reviewed to ensure that the treatments being paid for were indeed not part of the standard of care and were being pursued in lieu of evidence‐based treatment. Residence in the US was confirmed using the contact information provided for the campaign organizer and the information in the campaign text, which often included the residence of the beneficiary. Campaigns were excluded if the campaign organizer elected to leave the campaign description blank at the time of data collection. Ultimately, 163 campaigns did not confirm the residence of the beneficiary, 208 campaigns did not include a description, six campaigns were not raising money for ACT, and eight campaigns were found to be a duplicate of another. We therefore excluded 385 campaigns that did not meet eligibility criteria, leaving 615 campaigns in the final analysis.

Campaigns stating that the beneficiary intended to travel outside of the US to receive at least part of their ACT were classified in the “International travel” group, and if they did not, they were classified as “No international travel.” Campaigns for beneficiaries planning international travel included those that named clinics, cities, and countries to which they planned to travel to receive care and those that stated explicitly that they planned to travel outside of the US but did not mention a specific location.

### 2.2 Analysis

Demographic, clinical, and treatment information were extracted for the 615 campaigns. Variables included the individual's presenting gender, primary cancer type, cancer stage, and treatment modality. The diverse alternative cancer therapy modalities sought by the individuals represented by the campaigns were categorized into eight groups: “Pharmacologic and biologic” included supplements, antineoplaston, probiotics, insulin potentiation therapy, enemas, ozone, injections, and infusions; “Special diets” included extreme diets, such as organic, “raw foods”, Gerson diet, vegetarianism, etc.; “Traditional/Folk remedies” included traditional Chinese medicine, ayurvedic medicine, herbs and botanicals, homeopathy, and naturopathy; “Heat/Light/Sauna” included therapies meant to treat cancer using light and heat; “Hyperbaric oxygen” referred to treatments that often involved time in a hyperbaric chamber; “Mind–body therapies” included yoga, exercise, prayer, meditation, and deep breathing; “Bioelectromagnetic” therapy included treatments with magnets and electricity, including Rife therapy; and “Manual healing” included chiropractic manipulations and massage.

The demographic, clinical and treatment characteristics of the campaigns were described. Two‐sided Fisher's exact tests were used to examine associations between patient/campaign characteristics and plans for international travel. Two‐sided Mann–Whitney U tests were used to examine differences between the two groups in the campaign money requested, campaign money received, number of donors, and page shares. Kruskal–Wallis H tests were used to compare median campaign characteristics among treatment modalities. Statistical analyses were performed using Stata, version 16.1 (StataCorp), and RStudio, version 3.6.0 (R Foundation), and *p* < 0.05 was used as the threshold for significance. Flow mapping was performed using ArcGIS Online (Esri).

## 3 RESULTS

**FIGURE 1:**
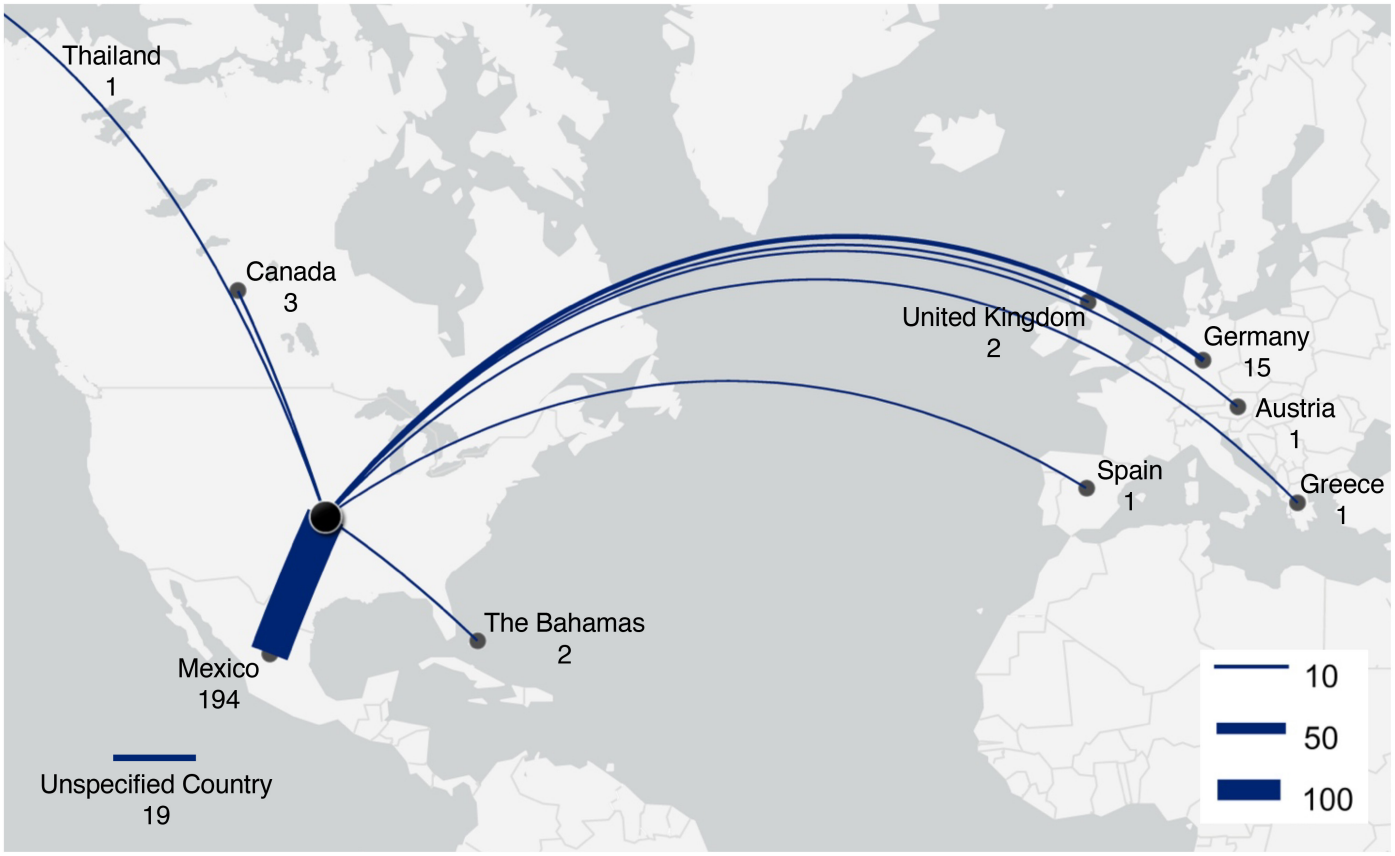
Intended treatment locations for GoFundMe® campaigns with plans for international travel.

Two hundred and thirty‐seven (38.5%) of the 615 campaigns mentioned plans to travel internationally for ACT (Table 1). Of these 237 campaigns, 194 (81.9%) specifically mentioned plans to travel to Mexico, 15 (6.3%) to Germany, 3 (1.3%) to Canada, 2 (0.8%) to The Bahamas, 2 (0.8%) to the United Kingdom, 1 (0.4%) to Austria, 1 (0.4%) to Greece, 1 (0.4%) to Spain, and 1 (0.4%) to Thailand (Figure 1). Nineteen campaigns (8.0%) mentioned international travel plans but did not specify a country. There were no significant differences between campaigns seeking support for international travel to obtain ACT and those that did not in terms of gender, cancer type, cancer stage, or country of residence (Table 1).

**TABLE 1:** Comparisons between GoFundMe® campaigns for unproven cancer treatments.

		International travel		
		No	Yes	*p‐*value
	All campaigns	*n* = 378	*n* = 237	
	*n* (%)	*n* (%)	*n* (%)	
Gender				
Female	380 (61.8%)	241 (63.8%)	139 (58.6%)	0.23
Male	235 (38.2%)	137 (36.2%)	98 (41.4%)	
Cancer type				
Breast	158 (25.7%)	93 (24.6%)	65 (27.4%)	0.51
Colorectal	63 (10.2%)	40 (10.6%)	23 (9.7%)	
Lung	39 (6.3%)	25 (6.6%)	14 (5.9%)	
Head & Neck	34 (5.5%)	20 (5.3%)	14 (5.9%)	
Brain	29 (4.7%)	22 (5.8%)	7 (3.0%)	
Pancreas	28 (4.6%)	16 (4.2%)	12 (5.1%)	
Esophagus/Gastric	27 (2.8%)	18 (4.8%)	9 (3.8%)	
Ovarian	23 (3.7%)	17 (4.5%)	6 (2.5%)	
Lymphoma	21 (3.4%)	10 (2.6%)	11 (4.6%)	
Bone & Soft tissue	18 (2.9%)	8 (2.1%)	10 (4.2%)	
Other^a^	175 (28.5%)	109 (28.8%)	66 (27.8%)	
Cancer stage				
IV	278 (45.2%)	159 (42.1%)	119 (50.2%)	0.14
I, II, or III	73 (11.9%)	49 (13.0%)	24 (10.1%)	
Unknown	264 (42.9%)	170 (45.0%)	94 (39.7%)	
Alternative medicine modality groups^b^				
Pharmacologic and Biologic	278 (45.2%)	157 (41.5%)	121 (51.1%)	0.02
Special diets	182 (29.6%)	104 (27.5%)	78 (32.9%)	0.17
Traditional/Folk	138 (22.4%)	90 (23.8%)	48 (20.2%)	0.32
Heat/Light/Sauna	63 (10.2%)	22 (5.8%)	41 (17.3%)	<0.001
Hyperbaric oxygen	60 (9.8%)	27 (7.1%)	33 (13.9%)	0.008
Mind–Body	59 (9.6%)	31 (8.2%)	28 (11.8%)	0.16
Bioelectromagnetic	20 (3.3%)	18 (4.8%)	2 (0.8%)	0.009
Manual healing	20 (3.3%)	12 (3.1%)	8 (3.4%)	1

*Note*: Percentages reported are within column (i.e., the denominator is the *n* for that column). The reported *p*‐values refer to comparisons between campaigns that included (yes) or excluded (no) mention of international travel.

^a^ Includes the following cancers: bile duct, bladder, cervix, endometrial, gallbladder, leukemia, liver, kidney, melanoma, multiple myeloma, neuroendocrine, prostate vulvar, thyroid, testicular, skin (non‐melanoma).

^b^ Groups are not mutually exclusive; one campaign could mention multiple CAM modalities, so the percentages do not add up to 100% in any of the columns.

Treatment modalities differed significantly between groups with heat/light/sauna therapies (17.3% vs. 5.8%, *p < 0*.001), hyperbaric oxygen therapy (13.9% vs. 7.1%, *p = 0*.008), and pharmacologic and biologic treatments (51.1% vs. 41.5%, *p = 0*.02) significantly more represented among campaigns with plans for travel outside the United States (Table 1). By contrast, bioelectromagnetic therapy was sought significantly more in the United States (4.8% vs. 0.8%, *p = 0*.009; Table 1). There were no significant differences in the frequency at which the two groups pursued special diets (*p = 0*.17), traditional and folk remedies (*p = 0*.32), mind–body therapies (*p = 0*.16), or manual healing (*p =* 1; Table 1). There were no significant differences in the median amount received, the median number of donations, or the median number of shares across campaigns fundraising for different treatment modality groups (Table S1). The median amount requested was significantly different by treatment modality group (Table S1).

Together, the 615 campaigns requested a total of $29,904,672 and raised a total of $6,637,730. Campaigns raising money for alternative therapies received internationally requested $17,417,039, and campaigns for alternative domestic treatments requested $12,487,633 (Table 2). The international travel campaigns requested significantly more money than the domestic campaigns on average (median: $35,000, interquartile range [IQR: $20,000 to $50,000] vs. $22,650 [IQR: $2392 to $45,750], *p < 0*.001; Table 2). Furthermore, the international travel campaigns raised significantly more than the domestic campaigns (median: $7833 [IQR: $3500 to $15,655] vs. $5035 [IQR: $2392 to $10,618], *p < 0*.001; Table 2) and had a higher average amount per donation than domestic campaigns (mean: $160.07 vs. $131.72). There was no significant difference between the two groups when it came to the percent of their fundraising goal they reached (*p = 0*.90); the international travel campaigns raised 29.7% of their fundraising goal (IQR: 10.0% to 57.6%), and domestic campaigns raised 29.9% of their fundraising goal (IQR: 11.3% to 56.9%; Table 2).

**TABLE 2:** Comparison of the financial and social engagement data between campaigns between GoFundMe® campaigns with plans for treatment abroad versus domestically.

	International travel		
	No	Yes	*p*‐value
	*n* = 378	*n* = 237	
Requested (USD)			
Sum	$12,487,633	$17,417,039	
Median, Interquartile range	$22,650 ($10,000 to $45,750)	$35,000 ($20,000 to $50,000)	<0.001
Received (USD)			
Sum	$3,459,022	$3,178,708	
Median, Interquartile range	$5035 ($2392 to $10,618)	$7833 ($3500 to $15,655)	<0.001
Percent of fundraising goal reached			
Median, Interquartile range	29.9% (11.3% to 56.9%)	29.7% (10.0% to 57.6%)	0.90
Donation deficit (USD)			
Median, Interquartile range	‐$13,436 (−$32,830 to −$3634)	‐$22,640 (−$37,710 to −$6385)	0.003
Number of unique donors			
Sum	26,260	19,858	
Median, Interquartile range	45 (24 to 89)	57 (27.25 to 114.50)	0.02
Number of page shares			
Sum	192,132	140,864	
Median, Interquartile range	290.5 (128 to 555)	377 (170 to 743)	0.008

Of the 615 campaigns, only 8.5% (*n* = 52) met or exceeded their fundraising goal; of those, 22 mentioned plans for international travel and 30 did not. The median difference between the fundraising goal and the actual amount donated was significantly larger for international campaigns than domestic campaigns (*p = 0*.003); the median campaign for international treatments had a financial shortfall of ‐$22,640 (IQR: ‐$37,710 to ‐$6385), and the median campaign for domestic treatments had a shortfall of ‐$13,436 (IQR: ‐$32,830 to ‐$3634; Table 2).

The campaigns in this study received donations from a total of 46,118 unique donors and were shared 332,996 times. When compared to campaigns for alternative domestic cancer treatments, campaigns planning international travel had significantly more unique donors (57 [IQR: 27.25 to 114.5] vs. 45 [IQR: 24 to 89], *p = 0*.02) and a significantly higher number of online page shares through the 10 forms of social media platforms linked with GoFundMe® (377 [IQR: 170 to 743] vs. 290.5 [IQR: 128 to 555], *p = 0*.008; Table 2).

## 4 DISCUSSION

Campaigns fundraising for international ACT requested and received significantly more money than campaigns seeking treatment domestically. They also had significantly more unique donors and were shared significantly more on social media, but there was no significant difference between the percent of fundraising goal reached by the two groups. Since campaigns mentioning international travel plans generally requested greater funds without an accompanying increase in the percent of fundraising goal received, international campaigns fell significantly shorter of their fundraising goals than campaigns without plans for international travel, creating greater unmet financial obligation and potential financial toxicity for the beneficiaries and their families. Prior studies have reported GoFundMe® fundraising data for both complementary therapies and ACT combined (Snyder et al., median requested: $19,880, median received: $5505.50; Song et al., median requested: $15,000, median received: $2870).^5^, ^15^ Our study of campaigns fundraising exclusively for ACT revealed higher median amounts requested ($30,000) and received ($5853) than both of these studies examining campaigns for both complementary therapies and ACT.

Given the substantial out‐of‐pocket cost of ACT, it is understandable that many individuals would seek help from others through online crowdfunding websites. Online crowdfunding encourages compelling personal narratives that convince both the beneficiary's real‐world community and strangers of the merit of their treatment plan and to donate to their cause.^16^, ^17^ Unfortunately, this leads many well‐meaning organizers and beneficiaries of campaigns for alternative treatments to exaggerate the efficacy of the therapies they are pursuing or to simply repeat the unsubstantiated claims of the clinics they hope to visit, which may perpetuate misinformation.^5^ More research on how misinformation claims within GoFundMe® campaigns affect donations and funding success would be beneficial to understanding if certain treatment‐related misinformation is used more frequently or is well received, as well as if such misinformation is more prominent in the public communication environment overall.

The social media engagement data from this study also gives cause for concern due to the spread of misinformation.^18^ Six hundred and fifteen campaigns for ACT were shared 332,996 times and received 46,118 unique donations. Campaigns including plans for international travel had significantly more engagement, both in terms of donors and number of page shares per campaign. It is not surprising that the group of international travel campaigns that requested significantly more money were also shared significantly more times on the ten social media platforms linked with GoFundMe®. The higher donation goals may have motivated the beneficiaries and organizers of these campaigns to broadcast to a wider audience of potential donors. Another possible explanation is that the narratives of the campaigns seeking ACT outside of the United States were somehow more compelling and therefore more likely to be shared by those who read them. Unfortunately, since GoFundMe® does not provide information about who shares a campaign, we could not determine if the sharing was done by a single individual (e.g., only the campaign organizer), or by multiple unique individuals who encountered the campaign separately. Since we did not include subsequent shares of the original link in our analysis and given that alternative therapy campaigns may not have used our search term “alternative cancer,” the current research likely underestimates reach.

The significantly larger number of unique donations to campaigns for alternative international treatments is interesting on several levels. First, it highlights that the financial cost associated with pursuing travel for ACT is borne not only by the individual but by their support network. Second, it did not lead to these campaigns reaching a significantly higher percent of their fundraising goal. Rather, since campaigns for international treatments were requesting significantly more money, the gap between their goal and the amount received was larger. In other words, although campaigns mentioning international travel received more money and a greater number of donations, they were ultimately less likely to secure the requested funds. This could strain a cancer patient's personal funds and create or amplify economic hardship. Previous reports have shown that patients seeking online crowdfunding for medical expenses frequently express an increased underlying financial need and request additional financial support including funds for rent payments, lost wages, food, and other urgent financial needs.^19^, ^20^ GoFundMe® campaign beneficiaries with the fewest financial resources and highest need have been shown to have the least successful crowdfunding campaigns.^19^, ^21^ This is further exacerbated in historically disadvantaged patients and those of socioeconomic disadvantage, who are often less successful in their fundraising campaigns because of the relative lack of community financial capacity, as has been previously reported.^19^, ^22^

In general, ACT is not covered by medical insurance, making it potentially financially toxic.^23^ Although the term “financial toxicity” has traditionally been used in the context of conventional cancer therapy, the financial burden of paying for ACT is just as relevant.^24^ A key part of minimizing financial toxicity is the promotion and pursuit of quality cancer care and the avoidance of low value treatment.^24^ Not only is ACT associated with high out‐of‐pocket costs, but it is also not evidence‐based cancer care.^23^, ^24^, ^25^ As these data demonstrate, campaigns that mention plans for international travel tend to demand significantly more money. While the campaigns in this study did not provide exact costs for each element of travel and treatment, it is noteworthy that campaigns for alternative international treatments, which made up 33.8% of the entire sample, requested 58.3% of the money. It is likely that the added cost of travel and lodging associated with receiving treatment in a foreign country may have contributed to these campaigns' higher donation goals. Taken together with the fact that campaigns for international treatments had a significantly larger financial shortfall, the decision to travel internationally for ACT indeed appears to exacerbate an already risky financial situation.

Each campaign included a body of text which campaign organizers used to explain their decision to decline conventional treatment in favor of ACT and frequently included references to their associated sources which advocated for ACT. The term “cancer misinformation” has previously been defined and includes recommendations to forego conventional cancer treatments, which is inherent in use of ACT.^26^, ^27^ It is therefore possible that these publicly shared campaign texts and their quoted sources may unwittingly increase the spread of health misinformation and its negative financial consequences. This effect is compounded by the significantly higher number of page shares among international campaigns, which extended the reach of these campaigns and their texts to a larger audience. The potential for crowdfunding sites, and campaigns for alternative international cancer treatments in particular, to indirectly serve as vectors of health misinformation warrants the attention of policy makers, researchers, medical providers and community leaders.

### 4.1 Limitations

The primary limitation of the study is that it relied on campaign texts written for the purpose of soliciting financial support for individuals seeking ACT. Information content was inconsistent across campaigns, and there is the possibility of misclassification bias since some campaigns may have been for individuals seeking ACT internationally but did not specify travel plans in their campaign. Additionally, the analysis did not determine whether the international travel plans as described in the campaigns were carried out. GoFundMe® campaign organizers have the option to delete an active or concluded campaign. It is possible that a campaign's success, failure, or conclusion could influence the choice to delete a posted campaign and consequently remove the campaign from our sample. The study is limited to GoFundMe® campaigns created between 2011–2019 and does not include campaigns created after COVID‐19 travel restrictions were lifted, which could limit generalizability to new and future campaigns. All campaigns came from the United States giving the data a US‐centric bias, and the inclusion criteria of English language limits generalizability among non‐English speaking populations. The term “alternative cancer” was used to potentially exclude any patients who are likely using non‐traditional therapies or mind–body therapies with their conventional cancer treatments, or otherwise defined as complementary medicine users. In the effort to exclude those receiving complementary medicines, we may not have evaluated all users of alternative cancer therapies. However, this highly specific search criteria resulted in the largest qualitative study, known to date, of patients traveling internationally for alternative cancer therapies.

## 5 CONCLUSIONS

Campaigns for patients seeking international travel for ACT received significantly more money, had significantly more donors, and were shared significantly more often on GoFundMe® than those seeking ACT domestically. ACT remains an attractive mode of cancer treatment, as evidenced both by the number of crowdfunding campaigns existing to support them and the amount of money donated to them. The data presented in this study demonstrate the potential financial toxicity that results for those seeking ACT, particularly those traveling internationally. This information can help researchers and cancer providers to target these issues for future research and cancer patient education.

## AUTHOR CONTRIBUTIONS

**John Peterson:** Formal analysis (equal); methodology (equal); writing – original draft (equal). **Trevor Wilson:** Formal analysis (equal); methodology (equal); writing – original draft (equal). **Melissa Watt:** Methodology (equal); writing – review and editing (equal). **Joshua D Gruhl:** Software (equal). **Sydney Davis:** Methodology (equal). **Jaxon Olsen:** Methodology (equal). **Matthew W. Parsons:** Methodology (equal). **Benjamin H Kann:** Writing – review and editing (equal). **Briony Swire‐Thompson:** Writing – review and editing (equal). **Angela Fagerlin:** Writing – review and editing (equal). **Echo L Warner:** Writing – review and editing (equal). **Andy King:** Writing – review and editing (equal). **Fumiko Chino:** Writing – review and editing (equal). **Skyler Johnson:** Conceptualization (lead); methodology (equal); supervision (lead); writing – review and editing (equal).

## FUNDING INFORMATION

Dr. Fumiko Chino is funded in part through the NIH/NCI Support Grant P30 CA008748.

## CONFLICTS OF INTEREST

The authors have no conflicts of interest to disclose.

## PRECIS

Among individuals fundraising online for alternative cancer therapies, those who mention plans to travel internationally appear to carry significantly greater financial risk. This group may be negatively impacted by not only the ineffectiveness of unproven or disproven cancer treatments but the potential financial toxicity on cancer patients and their families.

## Supporting information


Appendix S1.

Table S1.

## Data Availability

Data available on request from the authors.
