# Supplementary material for: International medical tourism of US cancer patients for alternative cancer treatments: Financial, demographic, and clinical profiles of online crowdfunding campaigns
Source: Cancer Med. 2023 Jan 19;12(7):8871–9. doi: 10.1002/cam4.5636 (PMC10134261; doi:10.1002/cam4.5636)
Supplement: Supplementary file 2 — Table S1. [file CAM4-12-8871-s002.docx]

Supplementary Table 1. Fundraising Characteristics Between Alternative Medicine Modality Groups

|  | **Number of Campaigns** | **Median Requested (USD) (p=.001)** | **Median Received (USD) (p=.20)** | **Median Number of Donations (p=.71)** | **Median Shares**  **(p=.91)** |
| --- | --- | --- | --- | --- | --- |
| Pharmacologic and Biologic | 278 | 30000 | 7510 | 61 | 349 |
| Special Diets | 182 | 25000 | 7101.5 | 55 | 349 |
| Traditional/Folk | 138 | 20000 | 6357.5 | 58 | 320.5 |
| Heat/Light/Sauna | 63 | 42000 | 8126 | 68.5 | 349 |
| Hyperbaric Oxygen | 60 | 34000 | 9716 | 68.5 | 385.5 |
| Mind-Body | 59 | 31000 | 7555 | 57 | 370.5 |
| Bioelectromagnetic | 20 | 30000 | 6200 | 56.5 | 279 |
| Manual Healing | 20 | 39500 | 8605 | 85 | 511 |

NOTES: Groups are not mutually exclusive; one campaign could mention multiple CAM modalities, so the number of campaigns by modality is greater than the total number of campaigns. Median modality group characteristics were compared using Kruskal-Wallis H tests.
